# Evaluation of Sex‐Based Differences in the Prescription of the Combination of Evidence‐Based Medicine After the Occurrence of an Acute ST‐Elevation Myocardial Infarction

**DOI:** 10.1002/clc.70195

**Published:** 2025-09-15

**Authors:** A. Evelo, E. Leegwater, W. J. R. Rietdijk, T. D. Warning, C. E. Schotborgh, L. E. Visser

**Affiliations:** ^1^ Department of Hospital Pharmacy Haga Teaching Hospital The Hague The Netherlands; ^2^ Department of Hospital Pharmacy Erasmus MC University Medical Center Rotterdam Rotterdam The Netherlands; ^3^ Department of Clinical Pharmacy Rijnstate Hospital Arnhem The Netherlands; ^4^ HagaAcademy & Innovation, Haga Teaching Hospital The Hague The Netherlands; ^5^ Department of Cardiology Haga Teaching Hospital The Hague The Netherlands

**Keywords:** evidence‐based medicine, secondary prevention, sex differences, ST‐elevation myocardial infarction, women

## Abstract

**Aim:**

To evaluate sex‐based differences in the prescription of the combination of evidence‐based medicine (cEBM) at discharge after an acute ST‐elevation myocardial infarction (STEMI). Secondly, we analysed risk factors for the absence of cEBM after discharge, and examined sex differences in adverse events.

**Methods:**

This retrospective cohort study compared women to men who were admitted with an acute STEMI at Haga Teaching Hospital between January 2017 and June 2023. The primary outcome was cEBM, defined as an active prescription of the combination of acetylsalicylic acid, P2Y_12_‐inhibitor, ACEi/ARB, beta‐blocker, and statin/ezetimibe on the day after discharge.

**Results:**

Among 1467 patients (27% women), women were older than men (74 years vs. 65 years, *p* < 0.001). cEBM was less often prescribed to women than men (66.0% vs. 72.8%, *p* = 0.013), primarily due to ACEi/ARB (82.1% vs. 87.7%, *p* = 0.007) and statins (90.2% vs. 95.2%, *p* = 0.001). In a multivariable logistic regression analysis, female sex was not associated with the absence of cEBM (Odds Ratio (OR) = 1.01, 95% confidence interval [95% CI]: 0.73–1.39). Other confounders such as increasing age, decreasing haemoglobin, and oral anticoagulants were correlated with the absence of cEBM.

**Conclusions:**

A smaller proportion of women were prescribed cEBM post‐STEMI compared to men. However, this difference disappeared when controlled for other confounders. Also, women remained to have a higher chance for a stroke or death at 6 months post‐discharge. These findings highlight the need for further research into sex disparities and their underlying confounders in the field of evidence‐based medicine after an acute STEMI.

## Summary

- Women did less often receive the combination of the five medicine after discharge.
- Not sex but other factors influenced the chance of the absence of the combination of the five medicine, such as age or the use of an oral anticoagulant.

## Graphical abstract

A smaller proportion of women were prescribed cEBM post‐STEMI compared to men. However, this difference disappeared when controlled for other confounders. Patient's age, haemoglobin, mcv, PCI during hospitalisation, CABG during hospitalisation, cardiogenic shock, LVEF, heart rate, systolic blood pressure, and oral anticoagulants remained in the final model as determinants for cEBM.

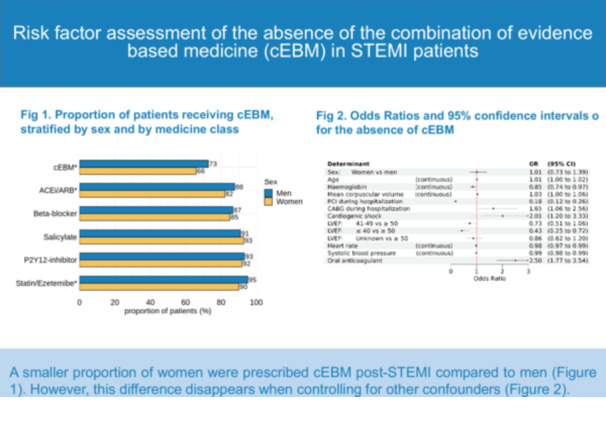

## 1 Introduction

Worldwide, cardiovascular disease is the leading cause of mortality and a major cause of morbidity, for both men and women [1]. The most severe type of acute coronary syndrome is acute ST‐elevation myocardial infarction (STEMI). Patients who have experienced STEMI benefit from pharmacological treatment to reduce the risk of subsequent ischemic events. This strategy is known as secondary prevention therapy. For five medicine classes, the independent clinical benefit is supported by evidence. The European and American guidelines recommend the combination of these five evidence‐based medicine (cEBM) groups which includes dual antiplatelet therapy (DAPT) (acetylsalicylic acid and a P2Y12‐inhibitor), lipid‐lowering therapy (statin and/or ezetimibe), a beta‐blocker, and an angiotensin‐converting enzyme inhibitor (ACEi) or angiotensinogen‐II receptor blocker (ARB) regardless of sex [2, 3]. This combination is recommended for all STEMI patients if not contra‐indicated.

Women with STEMI have a higher mortality rate than men [4, 5, 6, 7]. This could be attributed to differences between men and women in pathophysiology, clinical presentation and treatment [4, 7, 8, 9, 10, 11]. Moreover, women tend to receive less evidence‐based medicine post hospital discharge [4, 12, 13, 14]. Among studies, disparities are observed concerning which medicine classes (DAPT, statin, beta‐blocker, or ACEi/ARB) are less prescribed to women than men [4, 13, 15, 16, 17, 18]. However, literature about sex differences in cEBM defined as the combination of the five medicine groups after a STEMI is scarce, while the combination of drugs is associated with a reduced mortality risk [19, 20]. Just one Portuguese study reported on cEBM and showed that only 63.9% of the men versus 53.0% of the women were discharged with cEBM [13]. Although multiple studies described a difference in prescription of evidence based medicine between men and women [4, 13, 15, 16, 17, 18], sex disparities in cEBM at discharge has not yet been studied within the Dutch population. Moreover, determinants for cEBM in STEMI patients remain unknown and, thus, whether sex remains an independent factor in explaining the absence of cEBM is less clear.

Guidelines highlight that men and women equally benefit from therapies, thus, both sexes should be managed equally [21]. Moreover, it is shown that the risk of recurrent myocardial infarction (MI) and mortality decreases with every addition of a medicine class which highlights the need for cEBM [22]. As women remain to have a higher risk of adverse outcomes, it is of great relevance to determine whether sex or other determinants explain the disparity of cEBM between men and women. For this reason, our aim was to evaluate sex‐based differences in the prescription of cEBM at discharge after an acute STEMI. Secondly, we analysed risk factors for the absence of cEBM at discharge, and examined sex differences in mortality and revascularisation in patient sample of a large teaching hospital in The Netherlands.

## 2 Methods

### 2.1 Design and Study Population

A single‐centre, retrospective cohort study was conducted at the Haga Teaching Hospital in the Netherlands. All adult patients who were hospitalised and diagnosed with STEMI between January 2017 and July 2023 were screened for eligibility. Exclusion criteria were discharge to another hospital, death before discharge, registered refusal of using data for scientific research in the electronic patient file, and contraindications for one or more of the five medication groups, (Supporting Information Table 1). Contraindications for cEBM were a registered history of liver cirrhosis, angioedema, sick sinus syndrome, atrioventricular block, cholestasis, and rhabdomyolysis.

### 2.2 Outcomes

The primary study outcome was the incidence of cEBM in men and women. cEBM was defined as an active prescription of the combination of acetylsalicylic acid, P2Y12‐inhibitor, ACEi or ARB, beta‐blocker, and statin or ezetimibe on the day after discharge. If the prescription of one or more of these five therapeutic classes was absent in the electronic patient file, the patient was categorised as not being prescribed cEBM. Secondary outcomes were severe adverse events within 6‐months post discharge, namely: death, (non)STEMI, stroke, and revascularisation.

### 2.3 Data Source and Variables Collection

Data was extracted from the hospital's electronic patient records using the natural language processing tool and Clinical Data Collector, CTcue (IQVIA Patient Finder Solution‐CTcue B.V., Amsterdam, the Netherlands) [23]. Secondary outcomes and comorbidities for the absence of cEBM were identified by International Classification of Diseases and Related Health Problems 10th revision (ICD‐10) codes or the Dutch Diagnose Behandeling Combinatie (i.e. Diagnosis treatment combination) (DBC) codes (Supporting Information Tables S2–S4). In addition, retrieval from free‐text notes was used for all comorbidities as well as smoking.

Data on demographics were extracted on the first day of hospital admission and included sex and age. Additionally, on the first day of hospital admission we retrieved the medical history as documented in the electronic patient files. The following characteristics were identified as potential determinants for cEBM: hypertension, diabetes, dyslipidaemia, peripheral artery disease, dementia, cardiogenic shock, chronic kidney disease, previous Percutaneous Coronary Intervention (PCI), Coronary Artery Bypass Grafting (CABG), atrial fibrillation, coronary artery disease, stroke, (N)STEMI, heart failure, major bleeding and smoking status (defined as current, former or never). During hospitalisation the following variables were collected: left‐ventricular ejection fraction (LVEF) (categorized as ≥ 50, 41–49 and ≤ 40), Killip class, estimated glomerular filtration rate (eGFR) calculated with the CKD‐EPI equation, heart rate, systolic and diastolic blood pressure, haemoglobin and mean corpuscular volume and procedures during hospitalisation (PCI, CABG and coronary angiography). For eGFR, heart rate, systolic and diastolic blood pressure, haemoglobin, and mean corpuscular volume, we extracted the last measurement during hospitalisation. At the day post‐discharge, we reviewed if patients had a concurrent prescription of oral anticoagulants (OAC). Up to 6 months post‐discharge, we collected data on the occurrence of death, (N)STEMI, stroke, PCI, and CABG.

The process of data retrieval from free‐text notes for the comorbidities was validated by comparing the results of the text‐mining software with manual review in 10% of the total cohort. If more than 10% was false‐positive or false‐negative, the query was updated and checked once again until less than 10% was false‐positive or false‐negative.

### 2.4 Statistical Analysis

We analysed the data in three steps. First, descriptive statistics are used. Continuous variables were summarised by their mean and SD, or median and IQR depending on the normality of the variable. Normality was assessed using a Shapiro‐Wilk test. Categorical variables were summarised by counts and percentages. Comparisons between different strata were performed by Pearson's chi‐squared test for categorical variables, while an independent *t*‐test or a Mann–Whitney *U* test is applied dependent on the normality of the variable, respectively. Baseline characteristics describing the sample will be done using these statistics.

Second, for our primary outcome, that is cEBM, differences between men and women were described using descriptive statistics (counts and percentages). Further, counts and percentages for each of the cEBM medications were presented separately.

For our secondary outcomes, i.e., adverse events at 6‐months post hospital discharge, we used similar statistics as the descriptive statistics. For our other secondary outcome, i.e., factors associated with absence of cEBM at the day after hospital discharge we performed a multivariable logistic regression modelling with backwards deletion. Variables were included in the model if there was a clinically plausible association with cEBM. Variables were deleted using a backward deletion procedure based on Akaike information criterion (AIC) to create the final model. To determine the influence of sex on cEBM, sex was forced into the model. Variables were omitted from the analysis if more than 50% of the values were missing.

A sensitivity analysis was conducted to evaluate the impact of LVEF on cEBM. As mentioned before, LVEF was categorised into LVEF ≥ 50, LVEF 41–49, and LVEF ≤ 40. First, all patients were selected with LVEF ≥ 50 and cEBM was redefined to concomitant use of acetylsalicylic acid, P2Y12‐inhibitor, ACEi or ARB, and statin. Second, all patients were selected with LVEF ≤ 40 and the original cEBM was calculated. Thereafter, the effect of LVEF was explored on cEBM.

Statistical analyses have been performed using R, version 4.2.0. A *p*‐value < 0.05 was considered statistically significant.

## 3 Results

### 3.1 Baseline Characteristics

The study population consisted of 1467 patients with STEMI, of whom 1070 (73%) were men and 397 (27%) women. Women were older as compared to men (74 years vs. 65 years, *p* < 0.001), and had lower haemoglobin levels than men (8.0 vs. 8.9 mmol/l, *p* < 0.001). Women more often had hypertension and heart failure. In addition, a higher percentage of women had a prior stroke, and less often had an out‐of‐hospital cardiac arrest than men. During hospitalisation, they were less likely to undergo coronary angiography, PCI and CABG than men, see Table 1.

**Table 1:** Patient characteristics.

	Women (*n* = 397)	Men (*n* = 1070)	*p*‐value
**Baseline patient characteristics**			
Age (median [IQR])	74 [63–83]	65 [57–74]	
Smoking status (N, %)			< 0.001
Never	176 (44.3)	351 (32.8)	
Current	117 (29.5)	420 (39.3)	
Past	68 (17.1)	214 (20.0)	
Unknown	36 (9.1)	85 (7.9)	
Hypertension (N, %)	150 (37.8)	279 (26.1)	< 0.001
Diabetes (N, %)	69 (17.4)	155 (14.5)	0.198
Dyslipidaemia (N, %)	93 (23.4)	222 (20.7)	0.299
Peripheral artery disease (N, %)	24 (6.0)	40 (3.7)	0.075
Heart failure (N, %)	28 (7.1)	45 (4.2)	0.036
Atrial fibrillation (N, %)	25 (6.3)	52 (4.9)	0.335
Dementia (N, %)	7 (1.8)	4 (0.4)	0.016
Chronic kidney disease (N, %)	14 (3.5)	27 (2.5)	0.391
Prior myocardial infarction (N, %)	51 (12.8)	154 (14.4)	0.500
Previous PCI (N, %)	42 (10.6)	139 (13.0)	0.247
Previous CABG (N, %)	12 (3.0)	31 (2.9)	1.000
Previous coronary artery disease (N, %)	82 (20.7)	229 (21.4)	0.811
Prior stroke (N, %)	37 (9.3)	66 (6.2)	0.047
Prior major bleeding (N, %)	3 (0.8)	4 (0.4)	0.606
OAC co‐medicine (N, %)	62 (15.6)	181 (16.9)	0.606
Cardiogenic shock (N, %)	30 (7.6)	62 (5.8)	0.265
Out‐of‐hospital cardiac arrest (N, %)	24 (6.0)	103 (9.6)	0.039
**Characteristics during hospitalisation**			
Left ventricular ejection fraction (N, %)			0.161
≥ 50	112 (28.2)	299 (27.9)	
41–49	68 (17.1)	239 (22.3)	
≤ 40	39 (9.8)	96 (9.0)	
Unknown	178 (44.8)	436 (40.7)	
Killip Classification (N, %)			0.580
Class I	173 (93.0)	502 (95.3)	
Class II	6 (3.2)	13 (2.5)	
Class III	2 (1.1)	2 (0.4)	
Class IV	5 (2.7)	10 (1.9)	
Haemoglobine in mmol/L (median, IQR)	8.0 [7.3–8.5]	8.9 [8.3–9.5]	< 0.001
Mean Corpuscular Volume fl (median, IQR)	90 [87–93]	90 [87–93]	0.050
eGFR in ml/min/1.73 m² (median, IQR)	80 [62–91]	86 [70–96]	< 0.001
Heart rate in bpm (median, IQR)	77 [67–85]	73 [65–83]	< 0.001
Systolic blood pressure in mmHg (median, IQR)	121 [109–135]	123 [114–136]	0.010
Diastolic blood pressure in mmHg (median, IQR)	72. [66–80]	76.00 [69–83]	< 0.001
**Procedures during hospitalisation**			
PCI (N, %)	327 (82.4)	951 (88.8)	0.002
CABG (N, %)	22 (5.5)	153 (14.3)	< 0.001
Coronary angiography (N, %)	374 (94.2)	1047 (97.8)	0.001

*Note:* Characteristics of the included patients. Data are presented as *n* (%) or median (interquartile range).

Abbreviations: BPM, beats per minute; CABG, coronary artery bypass grafting; eGFR, estimated Glomerular Filtration Rate; IQR, interquartile range; (N)STEMI, (non‐) ST‐elevation myocardial infarction; OAC, oral anticoagulants; PCI, percutaneous coronary intervention.

### 3.2 Primary Outcome

**Figure 1:**
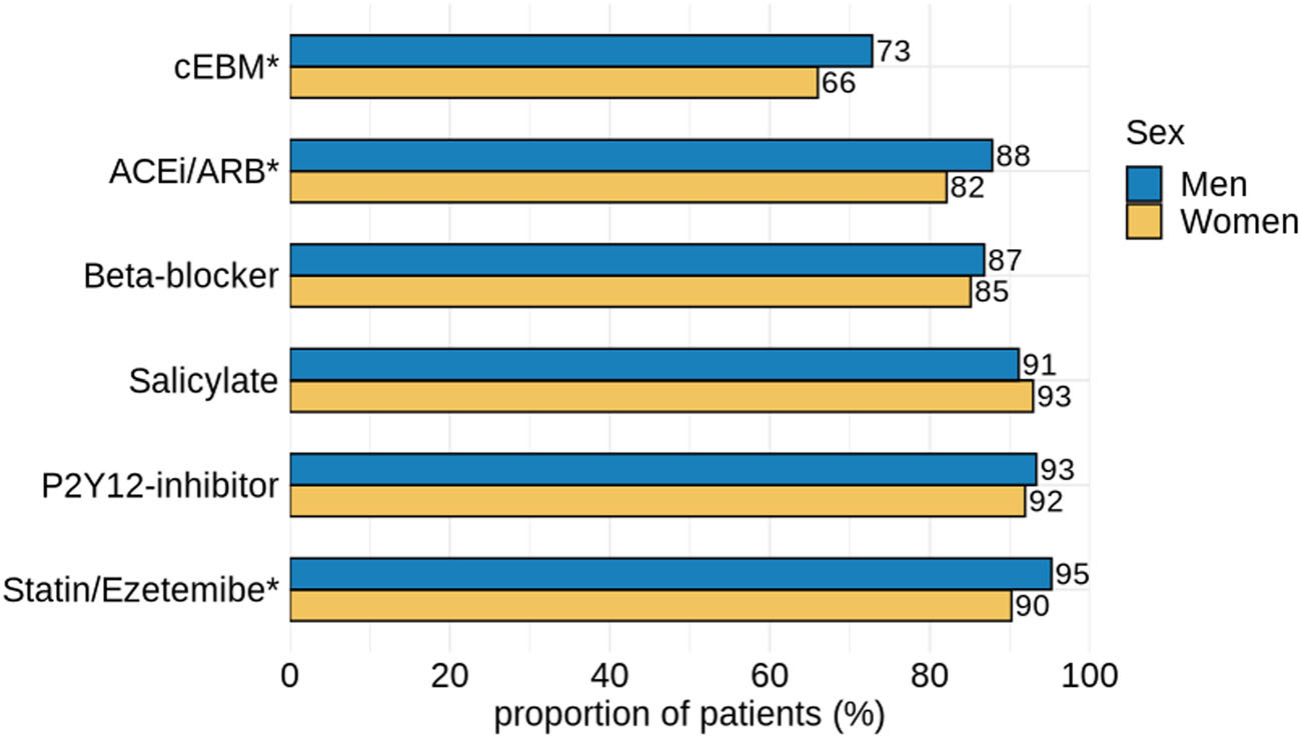
Proportion of patients receiving cEBM, stratified by sex and by medicine class. *: *p*‐value < 0.05. Abbreviations: ACEi, angiotensin‐converting enzyme inhibitor; ARB, angiotensinogen‐II receptor blocker; cEBM, combination of evidence‐based medicine.

Women were significantly less often prescribed cEBM post‐discharge than men (66.0% vs. 72.8%, *p* = 0.013). Specifically, ACEi or ARB (82.1% vs. 87.8% of men, *p* = 0.007) and statins or ezetimibe (90.2% vs. 95.2%, *p* < 0.001) were less often prescribed to women compared to men. Among women and men, non‐statistically significant differences were found in the prescription of acetylsalicylic acid, P2Y12 inhibitors and beta‐blockers at discharge, see Figure 1.

### 3.3 Secondary Outcomes & Sensitivity Analysis

The unadjusted 6 months mortality after STEMI was almost twice as high for women compared to men, (5.0% vs. 2.7%, *p* = 0.041). In addition, women more often had a stroke during the 6‐month follow‐up (5.5% vs. 2.9%, *p* = 0.024). However, no statistically significant sex differences were found in the 6‐month follow‐up for (N)STEMI and revascularisation, see Table 2. All above‐mentioned secondary outcomes were not adjusted for confounders.

**Table 2:** Adverse events within six months post‐discharge, unadjusted for confounders.

Adverse event	Women (*n* = 397)	Men (*n* = 1070)	*p*‐value
Mortality	20 (5.0)	29 (2.7)	0.041
New (N)STEMI	15 (3.8)	28 (2.6)	0.318
New stroke	22 (5.5)	31 (2.9)	0.024
Revascularisation	62 (15.6)	173 (16.2)	0.861
PCI	54 (13.6)	148 (13.8)	0.978
CABG	10 (2.5)	27 (2.5)	1.000

*Note:* Number of adverse events within six months post‐discharge. Data are presented as *n* (%).

Abbreviations: CABG, coronary artery bypass grafting; (N)STEMI, (non‐) ST‐elevation myocardial infarction; PCI, percutaneous coronary intervention.

**Figure 2:**
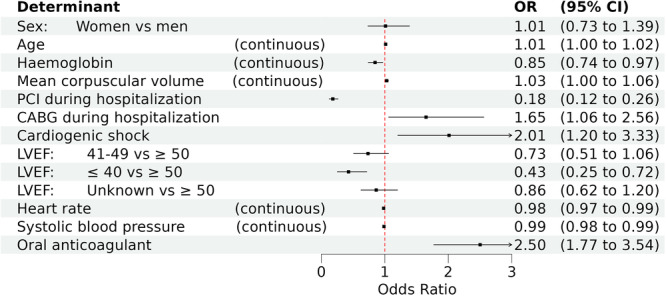
Odds Ratios (OR) and 95% confidence intervals (95% CI) for the absence of cEBM. For sex, the reference group is men. For smoking, the reference group is never. For left‐ventricular ejection fraction (LVEF), the reference group is LVEF ≥ 50.

Figure 2 shows the independent risk factors associated with the absence of cEBM and sex. Women did not have statistically significant different odds for the absence of cEBM than men (OR: 1.01, 95% CI: 0.73–1.39). Patient's age (OR: 1.01, 95% CI: 1.004–1.02), haemoglobin (OR: 0.85, 95% CI: 0.74–0.97), mean corpuscular volume (OR: 1.03, 95% CI: 1.004–1.06), PCI during hospitalisation (OR: 0.18, 95% CI: 0.12‐0.26), CABG during hospitalisation (OR: 1.65, 95% CI: 1.06–2.56), cardiogenic shock (OR: 2.01, 95% CI: 1.20–3.33), LVEF, heart rate (OR: 0.98, 95%CI: 0.97–0.99), systolic blood pressure (OR: 0.98, 95%CI: 0.98–0.99), and OAC co‐medicine (OR: 2.50, 95% CI: 1.77–3.54) remained in the final model as determinants for cEBM. Patients with LVEF ≤ 40 compared to LVEF ≥ 50 (OR: 0.43, 95% CI: 0.25–0.72), increasing heart rate, increasing systolic blood pressure, and patients who had a PCI during hospitalisation had higher odds of receiving cEBM than patients who did not have these characteristics. Contrary, increasing age, decreasing haemoglobin, increasing MCV, CABG, cardiogenic shock and OAC co‐medicine were associated with absence of cEBM.

Our sensitivity analysis in patients with LVEF ≥ 50 resulted in 67.9% of the women and 73.9% of the men receiving the four medicine treatment, a non‐statistically significant sex‐difference (*p* = 0.273). For LVEF ≤ 40, we also found a non‐statistically significant difference between the sexes as 74.4% of the women and 75.0% of the men received cEBM, (*p* = 0.909).

## 4 Discussion

In this retrospective cohort study, we examined sex differences and risk factors associated with cEBM following an acute STEMI in 1467 patients at a large teaching hospital in the Netherlands. Our findings indicate that approximately 70% of the patients were discharged with cEBM. Women were less often prescribed cEBM post‐STEMI (65.9% vs. 72.8% in men), due to less ACEi or ARB and lipid‐lowering therapy. However, this difference between women and men was not significant in a multivariable analysis adjusted for other background characteristics. Notably, increasing age, decreasing haemoglobin, increasing mcv, CABG, cardiogenic shock, and concurrent use of oral‐anticoagulants were associated with a higher risk of the absence of EBM. In contrast, LVEF ≤ 40 compared to LVEF ≥ 50, increasing heart rate, increasing systolic blood pressure, and PCI during hospitalisation were independent predictors for receiving EBM.

International guidelines recommend that all STEMI patients without a contraindication, should be discharged with low‐dose aspirin, a P2Y12‐inhibitor, a lipid‐lowering medicine, a beta‐blocker and an ACEi or ARB. Treatment should be individualised based on contraindications, possible drug interactions, and risks warranted for each treatment. Despite these guidelines, several studies reported underprescription of specific EBM classes [4, 12, 13, 18]. The aforementioned Portuguese study found that just over half of the patients received cEBM at discharge (53.0% for women vs. 63.9% for men) [13]. This Portuguese study included data from 10 diverse hospitals. Our study included data from a teaching hospital with a large cardiology department, which may be more adept at prescribing EBM, thus, possibly explaining our higher prescription rate. In addition, the Portuguese study did not exclude patients with contraindications for EBM, thus, possibly giving an overestimation of the absence of cEBM. The difference in proportion may also be due to increased prescription over time caused by improved implementation and adherence to guidelines [14, 24].

To optimise treatment, it is crucial to identify patient groups that are undertreated and understand the underlying reasons. Consistent with existing literature, we found that older age was a predictor for the absence of cEBM at discharge [12, 13]. Physicians may be hesitant to prescribe medicine to older patients due to concerns about side effects. However, guidelines do not recommend varying treatment based on age. Moreover, a recent study by Fayol et al. showed that patients aged 80 years and older had a reduced all‐cause mortality at 5 years when given high‐intensity lipid‐lowering therapy [25]. Thus, suggesting that lipid‐lowering treatment should not be denied based on older age. Although the age‐dependent inequality in EBM post‐STEMI has been reported in several studies, the issue persists [12, 13]. Future research should examine the motives and perceived barriers for the prescription of cEBM in elderly patients.

In addition to age, OAC‐using patients had a higher risk for the absence of EBM. The latest ESC guideline of 2023 recommends, as a default strategy, up to 1 week after ACS OAC + DAPT, followed by OAC + single antiplatelet therapy [2]. However, if the strategy is to reduce the ischemic risk, it is advised to give OAC + DAPT up to 1 month after ACS. Our study did not consider the time from STEMI to hospital discharge in determining cEBM. Therefore, some patients might still receive cEBM if they were prescribed OAC. As a sensitivity analysis, we excluded all patients with OAC as co‐medicine. Thereafter, 67.8% of the women and 75.6% of the men received cEBM at discharge (*p* = 0.002). Thus, OAC co‐medicine does not independently explain the absence of cEBM. Another factor which influenced cEBM, and probably the prescription of DAPT was haemoglobin. With every point haemoglobin level increases, the odds of receiving cEBM increased with 14%. This finding could possibly be explained by clinicians’ reluctance to prescribe antithrombotic therapy in cases of low haemoglobin.

Recently, a study showed that among patients with acute myocardial infarction who underwent early coronary angiography and had a preserved LVEF (≥ 50%), long‐term treatment with a beta‐blocker did not lead to a lower risk of death or a new myocardial infarction [26]. Thus, suggesting these patients do not have an indication for a beta‐blocker. Due to this recent information, we did a sensitivity analysis investigating the influence of the LVEF on cEBM, although during the time of our study, our patients were indicated to receive cEBM. Of our patients with a LVEF ≥ 50, around 30% did still not receive the combination of the four medicine treatment. LVEF ≤ 40 contributed to receiving cEBM. However, even in patients with LVEF ≤ 40, a quarter did not receive cEBM. Concluding, patients with LVEF ≤ 40 compared to patients with LVEF ≥ 50 has higher odds of cEBM.

Women were less likely to undergo coronary angiography, PCI, or CABG during admission. In addition, we found that PCI during hospitalisation was associated with a lower risk of the absence of cEBM. We hypothesise that after a PCI, physicians are more prone to prescribe dual antiplatelet therapy despite the fact that dual antiplatelet therapy is the default therapy after acute coronary syndrome. Although we found that women were less likely to undergo these procedures, we cannot assume there is a causal relationship with stroke or death at 6 months post‐discharge due to the retrospective nature of the research design. Moreover, 94% of the women and 98% of the men received CAG, with approximately 82% of women and 89% of men receiving PCI. Our rates were consistent with literature [27, 28]. We included patients based on the Dutch‐Diagnose‐treatment combination for STEMI, which is the Dutch system used to invoice hospital care. Various reasons can lead to withholding coronary angiography such as late presentation, patient's refusal of invasive procedure or contraindication for peri‐procedural anticoagulation.

As secondary outcome, we examined adverse events at 6 months post‐discharge. At 6 months post‐discharge, stroke occurred twice as often in women than in men. The same was the case for the mortality rate. The observed difference in mortality rates must be interpreted with caution, as we were unable to adjust for potential confounders. Our findings align with some previous studies [4, 14, 29, 30]. Other studies have reported that sex differences in mortality following STEMI no longer persisted after adjustment for confounders such as age and comorbidities [31, 32, 33]. The ongoing debate in regard to the role of sex in post‐STEMI mortality highlights the importance of proper adjustment. Importantly the adverse events rates reported in our study should not be attributed to the absence of cEBM, as no adjustments were made for any confounders (e.g. age or comorbidities).

Several limitations of our study should be acknowledged. The retrospective design means that the validity of the conclusions relies on the accuracy and completeness of the data in the electronic patient files. This could lead to an underreporting of all variables. However, we have no reason to believe that the documentation quality differed between men and women. Furthermore, we attempted to have included the important clinically relevant confounders, but cannot exclude that there are more confounders relevant that should be considered for future research. This study did not examine medicine adherence rates nor study the relation between the secondary outcomes and the absence of cEBM, and, thus, no implications on this subject can be made. At the same time it is necessary to state that the results are merely hypothesis generating by nature rather than hypothesis testing. The strength of our study is that we studied a large, real‐world data set to evaluate the prescribing patterns of physicians and their compliance with guidelines.

## 5 Conclusion

A smaller proportion of women were prescribed cEBM post‐STEMI compared to men. However, this difference seems due to other risk factor such as age, haemoglobin, and oral anticoagulants. Also women remained to have higher odds for a stroke or death at 6 months post‐discharge. These results signify the need for more studies on sex disparities and their underlying confounders related in the field of evidence‐based medicine after an acute STEMI.

## Ethics Statement

The study was performed in compliance with the principles of the Declaration of Helsinki, the International Conference on Harmonization‐Good Clinical Practice (ICH‐GCP) guidelines, and the laws and regulations of the Netherlands. Furthermore, this study is not within the scope of the Dutch law on Medical Research Involving Human Subjects (Dutch abbreviation: WMO). Therefore, we obtained a waiver for the WMO from the Medical Research Ethics Committee (MREC) of Leiden Den Haag Delft (METC‐number G21.126). The study was approved by the Institutional Scientific Review Board.

## Conflicts of Interest

The authors declare no conflicts of interest.

## Supporting information


**Supplementary table 1:** Numbers of patients per exclusion criteria. **Supplementary table 2:** variables and their respective DBC (diagnosis behandelcombinatie) codes. **Supplementary table 3:** variables and their respective ICD‐10 code. **Supplementary table 4:** variables and their respective Diagnosethesaurus code.

## Data Availability

The data underlying this article will be shared on reasonable request to the corresponding author.
