# Supplementary material for: Evaluation of Sex‐Based Differences in the Prescription of the Combination of Evidence‐Based Medicine After the Occurrence of an Acute ST‐Elevation Myocardial Infarction
Source: Clin Cardiol. 2025 Sep 15;48(9):e70195. doi: 10.1002/clc.70195 (PMC12434318; doi:10.1002/clc.70195)
Supplement: Supplementary file 1 — Supplementary table 1: Numbers of patients per exclusion criteria. Supplementary table 2: variables and their respective DBC (diagnosis behandelcombinatie) codes. Supplementary table 3: variables and their respective ICD‐10 code. Supplementary table 4: variables and their respective Diagnosethesaurus code. [file CLC-48-e70195-s001.docx]

Appendix

Supplementary table 4: Numbers of patients per exclusion criteria

| **Exclusion Criteria** | **Number of patients** |
| --- | --- |
| Liver cirrhosis | 8 |
| Angioedema | 17 |
| Sick sinus syndrome, | 4 |
| Atrioventricular block | 25 |
| Cholestasis | 19 |
| Rhabdomyolysis | 3 |
| Death before discharge | 0 |
| Discharge to another hospital | 1 |

Supplementary table 2: variables and their respective DBC (diagnosis behandelcombinatie) codes

| **variable** | **DBC discription** | **dbc -code** | **Specialty code description** | Specialty code |
| --- | --- | --- | --- | --- |
| dbc diagnose | ST elevatie hartinfarct | 204 | Cardiologie | 320 |
| diabetes | Diabetes mellitus chronisch pomptherapie | 223 | Interne geneeskunde | 313 |
| diabetes | Diabetes mellitus met secundaire complicaties | 222 | Interne geneeskunde | 313 |
| diabetes | Diabetes mellitus zonder secundaire complicaties | 221 | Interne geneeskunde | 313 |
| peripheral artery disease | P.A.O.D. 2, claudicatio intermittens | 418 | Chirurgie (heelkunde) | 303 |
| peripheral artery disease | P.A.O.D. 3, rustpijn | 419 | Chirurgie (heelkunde) | 303 |
| peripheral artery disease | P.A.O.D. 4, gangreen | 420 | Chirurgie (heelkunde) | 303 |
| peripheral artery disease | P.A.O.D. arm | 412 | Chirurgie (heelkunde) | 303 |
| atrial fibrillation | Atrium fibrilleren / flutter | 401 | Cardiologie | 320 |
| atrial fibrillation | Atriumfibrilleren, ritme- en geleidingsstoornis | 106 | Cardiologie | 320 |
| atrial fibrillation | Atriumfibrilleren, ritme- en geleidingsstoornis | 106 | Interne geneeskunde | 313 |
| coronary artery disease | Angina pectoris, onstabiel | 203 | Cardiologie | 320 |
| coronary artery disease | Angina pectoris, stabiel | 202 | Cardiologie | 320 |
| coronary artery disease | Follow-up na acuut coronair syndroom | 801 | Cardiologie | 320 |
| coronary artery disease | Non ST elevatie hartinfarct | 205 | Cardiologie | 320 |
| coronary artery disease | ST elevatie hartinfarct | 204 | Cardiologie | 320 |
| heart failure | Acuut hartfalen | 301 | Cardiologie | 320 |
| heart failure | Chronisch hartfalen | 302 | Cardiologie | 320 |
| myocardial infarction | Non ST elevatie hartinfarct | 205 | Cardiologie | 320 |
| myocardial infarction | ST elevatie hartinfarct | 204 | Cardiologie | 320 |
| stroke | Geen neurologie, werkdiagnose TIA (incl amaurosis fugax) | 9927 | Neurologie | 330 |
| stroke | Onbloedige beroerte | 1111 | Neurologie | 330 |
| stroke | TIA (inclusief amaurosis fugax) | 1112 | Neurologie | 330 |

Supplementary table 3: variables and their respective ICD-10 code

| **variable** | **ICD-10 description (in dutch)** | **ICD-10 code** |
| --- | --- | --- |
| Av-block | Tweedegraads atrioventriculair block | I44.1 |
| diabetes | Niet gespecificeerde diabetes mellitus | E14.5 |
| diabetes | Niet gespecificeerde diabetes mellitus | E14.4+ |
| diabetes | Niet gespecificeerde diabetes mellitus | E14.3+ |
| diabetes | Type 2 diabetes mellitus | E11.4+ |
| diabetes | Type 2 diabetes mellitus | E11.9 |
| hypertension | EssentiÃ«le (primaire) hypertensie | I10 |
| atrial fibrillation | Atriumfibrilleren en -flutter, niet gespecificeerd | I48.9 |
| atrial fibrillation | paroxismaal atriumfibrilleren | 39112 |
| atrial fibrillation | paroxismaal atriumfibrilleren | I48.0 |
| coronary artery disease | Acuut myocardinfarct, niet gespecificeerd | I21.9 |
| coronary artery disease | Acuut subendocardiaal myocardinfarct | I21.4 |
| coronary artery disease | Acuut transmuraal myocardinfarct van onderwand | I21.1 |
| coronary artery disease | Acuut transmuraal myocardinfarct van overige gespecificeerde lokalisaties | I21.2 |
| coronary artery disease | Instabiele angina pectoris | I20.0 |
| coronary artery disease | Vroeger myocardinfarct | I25.2 |
| heart failure | rechtszijdig hartfalen | 5237 |
| major bleeding | Overige gespecificeerde vormen van intracerebrale bloeding | I61.8 |
| myocardial infarction | Acuut subendocardiaal myocardinfarct | I21.4 |
| myocardial infarction | Acuut transmuraal myocardinfarct van onderwand | I21.1 |
| myocardial infarction | Vroeger myocardinfarct | I25.2 |
| stroke | 'Transient (cerebral) ischaemic attack' [TIA], niet gespecificeerd | G45.9 |
| stroke | Cerebraal infarct door niet gespecificeerde occlusie of stenose van cerebrale arteriÃ«n | I63.5 |
| stroke | Cerebraal infarct, niet gespecificeerd | I63.9 |
| stroke | Overige gespecificeerde 'transient (cerebral) ischaemic attacks' [TIA] en verwante syndromen | G45.8 |

Supplementary table 4: variables and their respective Diagnosethesaurus code

| **variable** | **Diagnosethesaurus** | **Diagnosethesaurus** |
| --- | --- | --- |
| Av-block | tweedegraads atrioventriculair blok | 0000005198 |
| chronic kidney disease | chronische nierinsufficiëntie stadium 3 | 0000061767 |
| chronic kidney disease | nierinsufficiëntie waarvoor chronische hemodialyse in instelling | 0000062001 |
| diabetes | diabetes mellitus type 2 | 0000002655 |
| dyslipidaemia | hypercholesterolemie | 0000035742 |
| dyslipidaemia | hyperlipidemie | 0000003345 |
| hypertension | essentiële hypertensie | 000005097 |
| hypertension | hypertensie in anamnese | 0000015751 |
| peripheral artery disease | perifeer arterieel vaatlijden Fontaine stadium 3 | 0000062672 |
| peripheral artery disease | verdenking op perifeer vaatlijden | 0000013292 |
| atrial fibrillation | atriumfibrilleren | 0000035644 |
| cabg | follow-up na coronary-artery bypass graft | 0000040250 |
| coronary artery disease | acuut inferolateraal myocardinfarct | 0000039039 |
| coronary artery disease | acuut myocardinfarct met ST-elevatie | 0000039040 |
| coronary artery disease | acuut myocardinfarct van inferoposterieure wand | 0000085629 |
| coronary artery disease | acuut myocardinfarct van laterale wand met ST-elevatie | 0000084675 |
| coronary artery disease | acuut myocardinfarct van onder-achterwand met ST elevatie | 0000089685 |
| coronary artery disease | acuut myocardinfarct van onderwand | 0000062072 |
| coronary artery disease | acuut myocardinfarct van voorwand | 0000039042 |
| pci | status na percutane coronaire interventie | 0000061724 |
| heart failure | acuut hartfalen | 0000039038 |
| heart failure | chronisch hartfalen | 0000039077 |
| stroke | cerebraal infarct | 0000005260 |
| stroke | cerebrovasculair accident | 0000005261 |
| stroke | cerebrovasculair accident met restverschijnselen in voorgeschiedenis | 0000058825 |
| stroke | ischemisch cerebrovasculair accident | 0000061514 |
| stroke | transient ischemic attack | 0000004164 |
